# Molecular and Biological Characterization of a New World Mono-/Bipartite Begomovirus/Deltasatellite Complex Infecting *Corchorus siliquosus*

**DOI:** 10.3389/fmicb.2020.01755

**Published:** 2020-07-23

**Authors:** Elvira Fiallo-Olivé, Jesús Navas-Castillo

**Affiliations:** Instituto de Hortofruticultura Subtropical y Mediterránea “La Mayora”, Consejo Superior de Investigaciones Cient ficas - Universidad de Málaga (IHSM-CSIC-UMA), Málaga, Spain

**Keywords:** geminiviruses, begomoviruses, DNA satellites, deltasatellites, *Corchorus siliquosus*, Malvaceae, virus taxonomy, phylogenetic analysis

## Abstract

The genus *Begomovirus* (family *Geminiviridae*) is the largest genus in the entire virosphere, with more than 400 species recognized. Begomoviruses are single-stranded DNA plant viruses transmitted by whiteflies of the *Bemisia tabaci* complex and are considered one of the most important groups of emerging plant viruses in tropical and subtropical regions. Several types of DNA satellites have been described to be associated with begomoviruses: betasatellites, alphasatellites, and deltasatellites. Recently, a family of single-stranded DNA satellites associated with begomoviruses has been created, *Tolecusatellitidae*, including the genera *Betasatellite* and *Deltasatellite*. In this work, we analyzed the population of begomoviruses and associated DNA satellites present in *Corchorus siliquosus*, a malvaceous plant growing wild in Central America, southeastern North America and the Caribbean, collected in Cuba. The genomes of isolates of two New World begomoviruses [(Desmodium leaf distortion virus (DesLDV) and Corchorus yellow vein Cuba virus (CoYVCUV)] and two deltasatellites [tomato yellow leaf distortion deltasatellite 2 (TYLDD2) and Desmodium leaf distortion deltasatellite (DesLDD)] have been cloned and sequenced from plants showing yellow vein symptoms. Isolates of one of the begomoviruses, CoYVCUV, and one of the deltasatellites, DesLDD, represent novel species. Experiments with infectious clones showed the monopartite nature of CoYVCUV and that DesLDD utilizes the bipartite DesLDV as helper virus, but not the monopartite CoYVCUV. Also, CoYVCUV was shown to infect common bean in addition to *Nicotiana benthamiana*. This is the first time that (i) a monopartite New World begomovirus is found in a host other than tomato and (ii) deltasatellites have been found in *C. siliquosus*, thus extending the host and helper virus ranges of this recently recognized class of DNA satellites.

## Introduction

Virus diseases that have emerged in the past three decades limit the production of important vegetable and fiber crops in tropical, subtropical, and temperate regions worldwide. Many of the causal viruses are transmitted by whiteflies (Hemiptera: Aleyrodidae), mainly by those belonging to the *Bemisia tabaci* cryptic species complex. Viruses known to be transmitted by whiteflies include members of the genera *Begomovirus*, *Crinivirus*, *Ipomovirus*, *Torradovirus*, and *Carlavirus* (Navas-Castillo et al., 2011), and two poleroviruses (genus *Polerovirus*) recently reported to be transmitted by *B. tabaci* (Ghosh et al., 2019; Costa et al., 2020).

The genus *Begomovirus* (family *Geminiviridae*) is the largest genus in the entire virosphere, with more than 400 species officially recognized. Begomoviruses are transmitted by whiteflies of the *Bemisia tabaci* complex (Navas-Castillo et al., 2011; Zerbini et al., 2017) and are considered among the most important emerging plant viruses. They infect a wide range of crops and wild plants in tropical and subtropical regions (Navas-Castillo et al., 2011). Begomoviruses are circular single-stranded DNA plant viruses with twin (geminate) quasi-icosahedral virions (Zerbini et al., 2017). Begomovirus genomes can be monopartite or bipartite depending on the presence of one or two (DNA-A and DNA-B) components, each of about 2.7 kb in size. Monopartite begomoviruses are widely spread in the Old World, but only a few examples have been reported from the New World. The genomes of monopartite begomoviruses resemble the DNA-A component. The DNA-A virion-sense strand encodes the coat and pre-coat proteins; the latter being present only in begomoviruses from the Old World. The DNA-A complementary-sense strand encodes the replication-associated protein (Rep), a transcriptional activator protein, a replication enhancer protein and C4 protein. DNA-B encodes a nuclear shuttle protein on the virion-sense strand and a movement protein on the complementary-sense strand. DNA-A and DNA-B share ∼200 nt in a common region (CR), located within the intergenic region that includes the replication origin. The Rep initiates viral DNA replication by binding to reiterated motifs (iterons) present in the CR and introducing a nick into the conserved non-anucleotide TAATATTAC. The CR shows a high level of nucleotide identity between both genome components of bipartite begomoviruses. Based on the phylogenetic analysis of full-length begomovirus genomic sequences (or DNA-A sequences), begomoviruses are classified into four lineages, Old World begomoviruses, New World begomoviruses, sweepoviruses and legumoviruses (Briddon et al., 2010).

Three types of DNA satellites have been described to be associated with begomoviruses, betasatellites (Briddon et al., 2003), alphasatellites (Briddon et al., 2004), and deltasatellites (Lozano et al., 2016). Recently, the International Committee on Taxonomy of Viruses (ICTV) modified the International Code of Virus Classification and Nomenclature to enable the classification of satellite nucleic acids (Adams et al., 2017). This change makes official the creation of a family of single-stranded DNA satellites associated with begomoviruses, *Tolecusatellitidae* (Briddon et al., 2016; Adams et al., 2017). The family *Tolecusatellitidae* includes two genera, *Betasatellite* and *Deltasatellite*. The genus *Betasatellite* includes DNA satellites about half the size of begomovirus genome components. Betasatellites encode the βC1 protein in the complementary-sense strand, which has important roles in symptom induction and suppression of transcriptional and post-transcriptional gene silencing (Zhou, 2013). Betasatellites have been found associated with monopartite begomoviruses in the Old World. Deltasatellites are non-coding DNA satellites associated with begomoviruses, of about a quarter size of begomoviral genome components (Fiallo-Olivé et al., 2012; Lozano et al., 2016). Tomato leaf curl deltasatellite, formerly ToLCV-sat, was the first DNA satellite identified in association with a plant virus, the monopartite Old World begomovirus tomato leaf curl virus (ToLCV) originating from Australia (Dry et al., 1997). Deltasatellites have been also found associated with bipartite New World begomoviruses infecting malvaceous weeds (Fiallo-Olivé et al., 2012, 2016) and sweepoviruses (Hassan et al., 2016; Lozano et al., 2016; Rosario et al., 2016). Also, the sequences of two uncharacterized deltasatellites from Philippines (KF433066) and India (AJ968684) have been deposited in GenBank. All deltasatellites contain common features; they share a small region with some sequence identity to a conserved region present in the betasatellites, an A-rich sequence, a predicted stem-loop structure containing the nonanucleotide TAATATTAC, and a predicted secondary stem-loop (Fiallo-Olivé et al., 2012; Lozano et al., 2016).

The genus *Deltasatellite* comprises 11 species (Briddon et al., 2016; Adams et al., 2017), five of them reported associated with bipartite New World begomoviruses. Until now, New World deltasatellites have been found only in the malvaceous plants *Malvastrum coromandelianum* and *Sidastrum micranthum* and only associated with two helper begomoviruses, Sida golden yellow vein virus and tomato yellow leaf distortion virus (Fiallo-Olivé et al., 2012, 2016). Recently, biological evidence has been obtained that New World deltasatellites depend on a limited range of begomoviruses for maintenance *in planta* (Fiallo-Olivé et al., 2016). In addition to the helper bipartite begomoviruses that the New World deltasatellites are associated with in nature, they can be transreplicated by the monopartite New World begomovirus tomato leaf deformation virus. In contrast, they cannot be maintained by the Old World begomoviruses tomato yellow leaf curl virus, tomato yellow leaf curl Sardinia virus and African cassava mosaic virus or the curtovirus beet curly top virus. New World deltasatellites do not affect the symptoms induced by the helper begomoviruses but in some instances they are able to reduce virus accumulation. Also, one New World deltasatellite has been shown to be transmitted by the whitefly *B. tabaci*, the natural vector of begomoviruses (Fiallo-Olivé et al., 2016).

In this study we analyzed the population of begomoviruses and associated DNA satellites present in wild *Corchorus siliquosus* plants collected in Cuba. The genomes of two New World begomoviruses (one bipartite and one monopartite) and two deltasatellites have been cloned and sequenced from plants showing yellow vein symptoms, the monopartite begomovirus and one of the deltasatellites representing novel species. Experiments with infectious clones showed that the monopartite begomovirus is also able to infect common bean and that the novel deltasatellite is maintained only by the bipartite begomovirus. This is the first time that a monopartite begomovirus native to the New World and deltasatellites have been found in *C. siliquosus*, extending the ranges of plant hosts and helper viruses for these recently recognized DNA satellites.

## Materials and Methods

### Plant Samples

**FIGURE 1:**
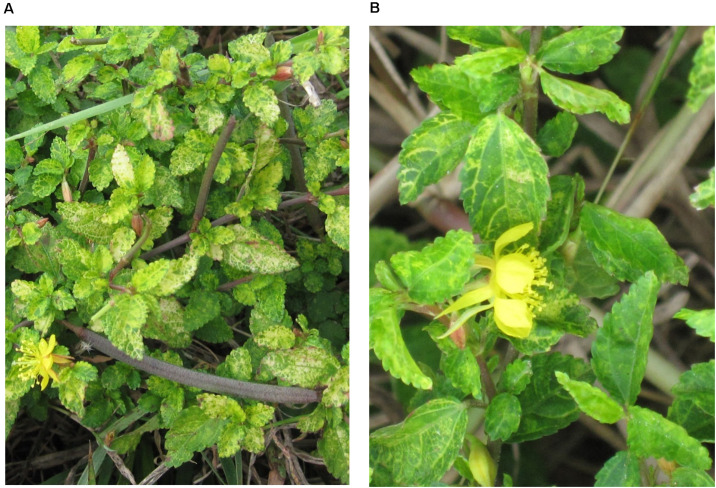
*Corchorus siliquosus* plants showing vein yellowing symptoms. **(A)** Sample 679. **(B)** Sample 705.

Leaves of *Corchorus siliquosus* plants (family Malvaceae) showing yellow vein symptoms (Figure 1) were collected in the province of Matanzas, Cuba, on December 2013 (Table 1). Each sample consisted of a few leaves which were dried, transported to the laboratory and held at 4°C. Morphological identification of the plants was confirmed at the molecular level by DNA barcoding using chloroplast rbcL and matK genes (Hollingsworth et al., 2009).

**TABLE 1:** Location of *Corchorus siliquosus* plants showing yellow vein symptoms sampled in the province of Matanzas, Cuba.

Sample	Municipality	Geographical coordinates
679	Matanzas	23°03.868′N 81°33.517′W
680	Matanzas	23°03.877′N 81°33.521′W
704	Jovellanos	22°53.152′N 81°17.163′W
705	Jovellanos	22°53.159′N 81°17.170′W
706	Jovellanos	22°53.167′N 81°17.178′W

### DNA Extraction and Cloning

Total DNA was extracted from leaf samples using a cetyltrimethylammonium bromide-based method (Permingeat et al., 1998) and then used as a template in rolling-circle amplification (RCA) using ϕ29 DNA polymerase (TempliPhi kit, GE Healthcare). Amplified RCA products were first digested with *Hpa*II, a 4-bp restriction enzyme, for preliminary restriction fragment length polymorphism (RFLP) analysis on 1.5% agarose gels. Then, RCA products were digested with a set of 6-bp restriction enzymes (*Eco*RI, *Bam*HI, *Hin*dIII, *Nco*I, *Sac*I). Putative full-length begomovirus genome components (∼2.6 kb) and deltasatellites (∼0.7 kb) obtained after digestion were size-selected and cloned into the appropriate sites of pBluescriptII SK(+) (Stratagene) or pGEM-T Easy Vector (Promega). Recombinant plasmid DNAs were transformed into *Escherichia coli* DH5α by electroporation and selected clones were sequenced in both directions by the Sanger method using M13 forward and reverse [for pBluescriptII SK(+)] or T7 promoter and SP6 promoter (for pGEM-T Easy Vector) primers and then by primer-walking (Macrogen Inc., Seoul, South Korea).

### Sequence Analysis

Sequences were assembled with SeqMan software included in the DNASTAR package (Lasergene). Open reading frames were identified using Open Reading Frame Finder (NCBI) and confirmed by a BLAST analysis^1^ of their deduced amino acid sequences. Initial sequence identity comparison was performed using the BLAST program, sequences were aligned with MUSCLE (Edgar, 2004), and pairwise identity scores were calculated using SDT (Sequence demarcation tool) (Muhire et al., 2014). *In silico* digestion analysis of cloned begomovirus and deltasatellite sequences was performed with SnapGene (Insightful Science, available at snapgene.com).

### Phylogenetic Analysis

For phylogenetic analysis of begomoviruses and deltasatellites, the maximum likelihood method was used after selecting the best-fit model of nucleotide substitution based on corrected Akaike Information Criterion and Bayesian Information Criterion as implemented in MEGA 7 (Kumar et al., 2016). For analysis of begomoviruses, the most closely related DNA-A and DNA-B components were selected as well as sequences belonging to different clades of New World begomoviruses. For phylogenetic analysis of deltasatelites, the deltasatellites isolates described in this work and one isolate belonging to each deltasatellite species (Briddon et al., 2016; Adams et al., 2017) were included.

### Recombination Analysis

Recombination analysis of sequences reported in this study was performed using RDP4 (Martin et al., 2015) after alignment with MUSCLE (Edgar, 2004). Only recombination events detected with at least five methods were considered. To carry out the recombination analysis of the begomoviruses, the sequences with highest SWeBLAST scores (window size of 200 and step size of 200) were selected (Fourment et al., 2008). For deltasatellites, all the sequences available in GenBank were included in the analysis.

### Construction of Infectious Clones

Infectious dimeric clones of the begomovirus genomic components and one of the deltasatellites characterized in this work were constructed as previously described (Fiallo-Olivé et al., 2016). Inserts from monomeric clones of each viral component or deltasatellite were released from the plasmids, religated and subjected to RCA. RCA products were partially digested to produce dimeric molecules which were subsequently cloned in a plasmid vector. The inserts of the dimeric clones were excised and subcloned in the binary vector pCAMBIA0380. Details of the restriction enzymes and vectors used in each cloning step are given in Supplementary Table 1. Ligation reactions were transformed into *Escherichia coli* DH5α by electroporation (25 μF, 200 Ω, 2500 V) in a Gene Pulser XCell (Bio-Rad). In each cloning step, clones were verified by digestion. Clones in the binary vector with inserts of the expected size were sequenced (Macrogen Inc., Seoul, South Korea) and transformed into *Agrobacterium tumefaciens* C58C1 by electroporation (25 μF, 200 Ω, 2500 V).

### Plant Inoculation and Begomovirus and Deltasatellite Detection

For agroinoculation assays, *A. tumefaciens* cultures harboring each dimeric construct were grown and inoculated as previously described (Fiallo-Olivé et al., 2016). Plant species used for agroinoculation assays were *Nicotiana benthamiana*, *Corchorus olitorius* (seeds purchased from B&T World Seeds), common bean (*Phaseolus vulgaris*) cv. Donna, tomato (*Solanum lycopersicum*) cv. Moneymaker, pepper (*Capsicum annuum*) cv. California Wonder and zucchini (*Cucurbita pepo*) cv. Milenio. Plants were inoculated with *A. tumefaciens* cultures containing clones of viral DNA components and deltasatellites by stem puncture inoculation. *N. benthamiana* plants were inoculated at the four-leaf stage and the rest of the plants at the two-leaf stage. Plants were maintained in an insect-free growth chamber (25°C during the day and 18°C at night, 70% relative humidity, with a 16-h photoperiod at 250 μmol s^–1^ m^–2^ of photosynthetically active radiation) until analyzed. All agroinoculation experiments were repeated twice and each contained 12 plants, with the exception of Exp. 2 with *C. olitorius* in which eight plants were inoculated. Plants that served as negative controls were mock inoculated with *A. tumefaciens* C58C1 cultures containing empty vector.

For biolistic inoculation, the monomeric clone of CoYVCuV obtained in this work was used to inoculate *N. benthamiana* and *C. olitorius* plants. Viral DNA insert was released from the vector by digestion with *Sac*I, circularized using T4 DNA ligase (Roche Diagnostics) and then subjected to RCA. The RCA product was precipitated onto 1.0 μm gold microcarriers (Bio-Rad) which were resuspended in ethanol. Plants were inoculated with a “Bim-Lab” instrument (Bio-Oz Biotechnologies). Both third and fourth leaves of each plant were shot twice with 5 μL of the RCA product-coated microcarriers. Biolistic inoculation experiments were repeated twice and each contained 12 (*N. benthamiana*) or 10 (*C. olitorius*) plants. Plants that served as negative controls were mock inoculated with the microcarriers in ethanol.

Apical leaves were used for tissue blots of petiole cross-sections (tissue printing) performed on positively charged nylon membranes (Roche Diagnostics) at 33 days post-inoculation. In the case of *N. benthamiana*, stem cross-sections were employed instead. Hybridization was carried out using digoxigenin-labeled DNA probes specific to each viral genomic component and deltasatellites. The probes were prepared by PCR with primers specifically designed for each begomovirus genomic component and one of the deltasatellites (Supplementary Table 2) according to the DIG-labeling detection kit (Roche Diagnostics). Hybridization was carried out under high stringency conditions [washing steps at 65°C in 0.1× SSC (15 mM NaCl, 1.5 mM sodium citrate) and 0.1% sodium dodecyl sulfate] following standard procedures. Hybridization signals were detected on X-ray film after treatment with CDP-Star (Roche Diagnostics).

## Results

### A Bipartite and a Monopartite Begomovirus, the Latter Representing a Novel Species, Infect *Corchorus siliquosus* in Cuba

Digestion with *Hpa*II of RCA-amplified products from the five *C. siliquosus* leaf samples yielded restriction patterns (Supplementary Figure 1) supporting the suspected begomovirus infections based on the symptomatology observed in the field, consisting of yellow veins (Figure 1). The complexity of the restriction patterns was suggestive of mixed infections. Sequencing of cloned DNA fragments following digestion with 6-bp enzymes confirmed the presence of DNA-A and DNA-B genomic components in the five samples. In total, 15 DNA-A and 21 DNA-B full-length clones were sequenced (Table 2). All of them showed the organization of typical New World begomoviruses.

**TABLE 2:** Begomoviruses and deltasatellites isolated from *Corchorus siliquosus* plants showing yellow vein symptoms sampled in the province of Matanzas, Cuba.

Sample	Begomovirus/deltasatellite	Genome component	Isolate	Restriction enzyme	Size (nt)	GenBank acc. no.
679	DesLDV	DNA-A	CU-Co679-1-13	*Sac*I	2569	MF773881
	DesLDV	DNA-A	CU-Co679-2-13	*Bam*HI	2570	MF773882
	DesLDV	DNA-B	CU-Co679-1-13	*Sac*I	2497	MF773891
	DesLDV	DNA-B	CU-Co679-2-13	*Sac*I	2497	MF773892
680	DesLDV	DNA-A	CU-Co680-1-13	*Sac*I	2570	MF773883
	DesLDV	DNA-A	CU-Co680-2-13	*Sac*I	2570	MF773884
	DesLDV	DNA-A	CU-Co680-3-13	*Sac*I	2569	MF773885
	DesLDV	DNA-B	CU-Co680-1-13	*Sac*I	2498	MF773893
	DesLDV	DNA-B	CU-Co680-2-13	*Sac*I	2497	MF773894
	DesLDV	DNA-B	CU-Co680-3-13	*Eco*RI	2498	MF773895
	DesLDV	DNA-B	CU-Co680-4-13	*Eco*RI	2498	MF773896
	DesLDV	DNA-B	CU-Co680-5-13	*Eco*RI	2498	MF773897
	ToYLDD2		CU-Co680-S8-13	*Sac*I	692	MF773917
	ToYLDD2		CU-Co680-S11-13	*Sac*I	692	MF773918
	ToYLDD2		CU-Co680-S17-13	*Sac*I	692	MF773919
704	DesLDV	DNA-A	CU-Co704-1-13	*Sac*I	2570	MF773886
	DesLDV	DNA-A	CU-Co704-2-13	*Sac*I	2570	MF773887
	DesLDV	DNA-B	CU-Co704-1-13	*Hin*dIII	2499	MF773898
	DesLDV	DNA-B	CU-Co704-2-13	*Hin*dIII	2499	MF773899
	DesLDV	DNA-B	CU-Co704-3-13	*Hin*dIII	2499	MF773900
	DesLDV	DNA-B	CU-Co704-4-13	*Sac*I	2498	MF773901
	DesLDD		CU-Co704-H1-13	*Hin*dIII	666	MF773920
	DesLDD		CU-Co704-H6-13	*Hin*dIII	666	MF773921
	DesLDD		CU-Co704-H7-13	*Hin*dIII	666	MF773922
	DesLDD		CU-Co704-H8-13	*Hin*dIII	666	MF773923
	DesLDD		CU-Co704-H9-13	*Hin*dIII	666	MF773924
	DesLDD		CU-Co704-H10-13	*Hin*dIII	666	MF773925
705	DesLDV	DNA-A	CU-Co705-1-13	*Sac*I	2569	MF773888
	DesLDV	DNA-B	CU-Co705-1-13	*Eco*RI	2531	MF773902
	DesLDV	DNA-B	CU-Co705-2-13	*Sac*I	2530	MF773903
	DesLDV	DNA-B	CU-Co705-3-13	*Eco*RI	2531	MF773904
	DesLDV	DNA-B	CU-Co705-4-13	*Sac*I	2530	MF773905
	DesLDV	DNA-B	CU-Co705-5-13	*Eco*RI	2531	MF773906
	DesLDV	DNA-B	CU-Co705-6-13	*Sac*I	2531	MF773907
	DesLDV	DNA-B	CU-Co705-7-13	*Sac*I	2530	MF773908
	DesLDV	DNA-B	CU-Co705-8-13	*Eco*RI	2531	MF773909
	CoYVCUV		CU-Co705-1-13	*Eco*RI	2576	MF773912
	CoYVCUV		CU-Co705-2-13	*Eco*RI	2576	MF773913
	CoYVCUV		CU-Co705-3-13	*Sac*I	2576	MF773914
	CoYVCUV		CU-Co705-4-13	*Sac*I	2576	MF773915
	DesLDD		CU-Co705-H1-13	*Hin*dIII	666	MF773926
	DesLDD		CU-Co705-H2-13	*Hin*dIII	665	MF773927
	DesLDD		CU-Co705-H3-13	*Hin*dIII	666	MF773928
	DesLDD		CU-Co705-H4-13	*Hin*dIII	665	MF773929
	DesLDD		CU-Co705-H5-13	*Hin*dIII	665	MF773930
	DesLDD		CU-Co705-H6-13	*Hin*dIII	665	MF773931
	DesLDD		CU-Co705-H7-13	*Hin*dIII	665	MF773932
	DesLDD		CU-Co705-H8-13	*Hin*dIII	665	MF773933
	DesLDD		CU-Co705-H11-13	*Hin*dIII	666	MF773934
706	DesLDV	DNA-A	CU-Co706-1-13	*Sac*I	2570	MF773889
	DesLDV	DNA-A	CU-Co706-2-13	*Sac*I	2570	MF773890
	DesLDV	DNA-B	CU-Co706-1-13	*Sac*I	2530	MF773910
	DesLDV	DNA-B	CU-Co706-2-13	*Sac*I	2531	MF773911
	CoYVCUV		CU-Co706-1-13	*Sac*I	2576	MF773916

*DesLDV, Desmodium leaf distortion virus; CoYVCUV, Corchorus yellow vein Cuba virus; ToYLDD2, tomato yellow leaf distortion deltasatellite 2, DesLDD; Desmodium leaf distortion deltasatellite.*

**FIGURE 2:**
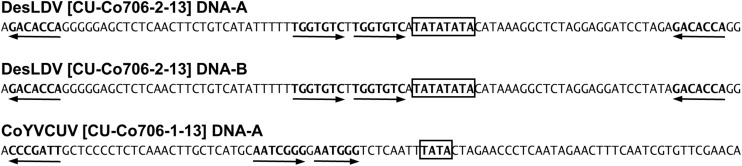
Iterons (arrows) of genome components of begomoviruses Desmodium leaf distortion virus (DesLDV) and Corchorus yellow vein Cuba virus (CoYVCUV). TATA motifs of the Rep promoter are highlighted with boxes.

**FIGURE 3:**
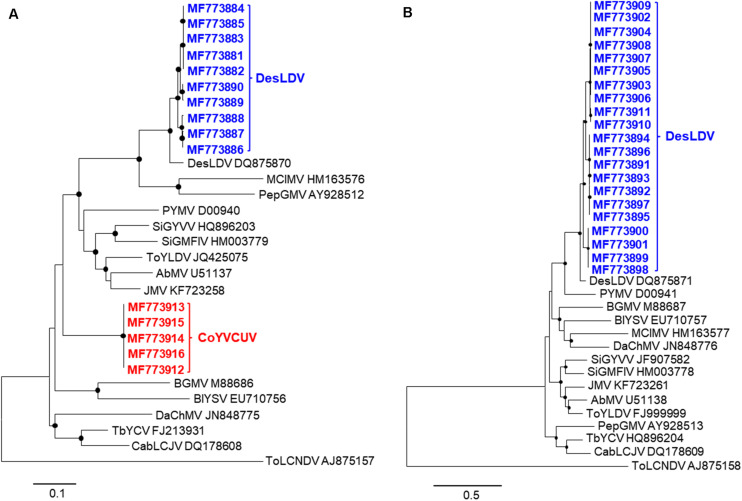
Phylogenetic trees illustrating the relationships of isolates of Desmodium leaf distortion virus (DesLDV) DNA-A **(A)** and DNA-B **(B)** (bold in blue) and Corchorus yellow vein Cuba virus (CoYVCUV) DNA-A (bold in red) isolates with other New World begomoviruses. The trees were constructed by the maximum likelihood method (1000 replicates) with the MEGA 7 program using the best fit model, TN93 + G + I for DNA-A and HKY + G for DNA-B. AbMV, Abutilon mosaic virus; BGMV, bean golden mosaic virus; BlYSV, Blainvillea yellow spot virus; CabLCJV, cabbage leaf curl Jamaica virus; DaChMV, Dalechampia chlorotic mosaic virus; JMV, Jatropha mosaic virus; MClMV, melon chlorotic mosaic virus; PepGMV, pepper golden mosaic virus; PYMV, potato yellow mosaic virus; SiGMFlV, Sida golden mosaic Florida virus; SiGYVV, Sida golden yellow vein virus; TbYCV, tobacco yellow crinckle virus; ToYLDV, tomato yellow leaf distortion virus. The Old World begomovirus tomato leaf curl New Delhi virus (ToLCNDV) was used as an outgroup. The bars below the trees indicate the number of nucleotide substitutions per site. Nodes with bootstrap values higher than 50% are marked with filled circles.

Pairwise comparison analysis carried out with SDT showed that 10 of the DNA-As (isolated from the five samples as *Bam*HI or *Sac*I fragments, GenBank acc. no. MF773881-MF773890) (Table 2) showed a nucleotide identity of 97.3–100% between them and the highest identity (94.1–94.3%) with Desmodium leaf distortion virus (DesLDV) DNA-A (DQ875870). In accordance with the current taxonomic guidelines for the genus *Begomovirus* (a new DNA-A sequence with ≥91% pairwise identity to an available begomovirus DNA-A sequence will belong to the same species) (Brown et al., 2015), the isolates described here belong to the species *Desmodium leaf distortion virus*. DesLDV is a bipartite New World begomovirus previously found infecting only the wild fabaceous *Desmodium glabrum* in Yucatán, Mexico (Hernández-Zepeda et al., 2009). DNA-Bs (also isolated from the five samples as *Eco*RI, *Hin*dIII or *Sac*I fragments, MF773891-MF773911) (Table 2) showed nucleotide identity of 90.2–100% between them and the highest identity (87.9–88.9%) with DesLDV DNA-B from Mexico (DQ875871). A common region of 161 nt was identified in the cloned DNA-As and DNA-Bs of DesLDV which showed an identity of 93.8–96.9% between both components. Also, four copies of identical iterons, two of them inverted, were present in both genome components (Figure 2), thus supporting that they constitute cognate pairs. Phylogenetic analysis showed that both DNA-A and DNA-B components of DesLDV isolates cloned from *C. siliquosus* plants, as expected, grouped with the DesLDV isolate from *D. glabrum* characterized in Mexico (Hernández-Zepeda et al., 2009; Figure 3). Recombination analysis of a representative isolate of DesLDV (Cuba-Corchorus 706-2-2013), showed the recombinant nature of the DNA-A. Interestingly, the major parent of the only recombination event detected is Corchorus yellow spot virus, a begomovirus isolated from *C. siliquosus* in Mexico (Hernández-Zepeda et al., 2007) and the minor parent an isolate of cabbage leaf curl virus from Florida (Abouzid et al., 1992; Table 3). No recombination events were detected in the DNA-B.

**TABLE 3:** Recombinant fragments detected in the DNA-A sequences of Desmodium leaf distortion virus – (Cuba-Corchorus 706-2-2013) (DesLDV) and Corchorus yellow vein Cuba virus – (Cuba-Corchorus 706-1-2013) (CoYVCUV) by at least five methods included in the RDP4 package.

Recombinant virus	Recombination breakpoints	Parent-like sequences		*p*-value	Methods that detected recombination^a^
		Major	Minor		
DesLDV	1917–2561	CoYSV DQ875868	CabLCV U65529	7.224 × 10^–58^	R, G, B, M, C, S, 3S
CoYVCUV	68–262	Unknown	DesLDV DQ875870	7.330 × 10^–15^	R, G, B, M, C, S
CoYVCUV	1186–1281	JMV KJ174331	DaChMV JN848775	3.841 × 10^–6^	R, G, B, S, 3S
CoYVCUV	1416–1605	JMV KJ174331	DaChMV JN848775	3.818 × 10^–10^	R, G, B, S, 3S

*^a^The method with the lower p-value obtained for each fragment is underlined. CabLCV, cabbage leaf curl virus; CoYSV, Corchorus yellow spot virus; DaChMV, Dalechampia chlorotic mosaic virus; JMV, Jatropha mosaic virus. R, RDP; G, GENCONV; B, BootScan; M, MaxChi; C, Chimaera; S, SiScan; 3S, 3Seq.*

The other five full-length DNA-A components (isolated from samples 705 and 706 and cloned as *Eco*RI or *Sac*I fragments, MF773912-MF773916) (Table 2) showed an identity of 99.8–100% between them and only 76.6–77.4% with the cloned DesLDV isolates. The highest identity (81.9%) was with the DNA-A of two begomoviruses from the Caribbean, tobacco yellow crinkle virus (FJ213931) (Fiallo-Olivé et al., 2009) and Jatropha mosaic virus (KJ174331) (Melgarejo et al., 2015). In accordance with the above-mentioned taxonomic guidelines for the genus *Begomovirus*, the isolates described here represent a novel species for which we propose the name *Corchorus yellow vein Cuba virus* (CoYVCUV). Phylogenetic analysis showed that the DNA-As of CoYVCUV isolates were highly divergent and grouped in a single cluster with a high bootstrap value, thus supporting that this begomovirus constitutes a novel species (Figure 3A). Recombination analysis of a representative CoYVCUV DNA-A (Cuba-Corchorus 706-1-2013), also showed its recombinant nature. The three recombination events detected involved begomoviruses infecting wild plants, Jatropha mosaic virus, Dalechampia chlorotic mosaic virus and DesLDV from Dominican Republic, Venezuela and Mexico, respectively (Hernández-Zepeda et al., 2009; Fiallo-Olivé et al., 2013; Melgarejo et al., 2015; Table 3).

**FIGURE 4:**
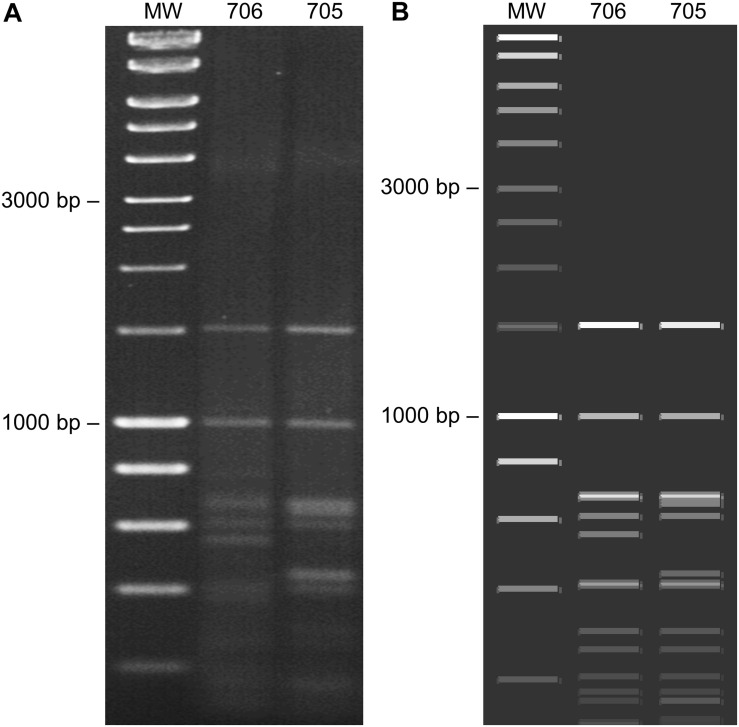
Restriction fragment length polymorphism (RFLP) analysis of begomoviruses and deltasatellites infecting *Corchorus siliquosus* (samples 705 and 706). **(A)** RFLP performed by digestion of rolling circle amplification (RCA) products obtained from DNA extracts with the restriction enzyme *Hpa*II revealed on a 1.5% agarose gel. **(B)** *In silico* RFLP analysis (*Hpa*II) of the begomovirus and deltasatellite sequences obtained from each sample, performed with SnapGene (Insightful Science, available at snapgene.com). MW: molecular weight marker (HyperLadder 1 kb, Bioline).

The CR of the DesDLDV DNA-B and the equivalent region of the CoYVCUV DNA-A present in the same samples showed a low identity (65.8–68.0%) and dissimilar iterons (Figure 2). Based on this, the DNA-A of CoYVCUV and the DNA-B of DesLDV would not constitute a cognate pair. On the other hand, although the DNA-A of CoYVCUV showed the typical genome organization of a bipartite New World begomovirus DNA-A, no DNA-B could be found for this virus in the two samples analyzed despite repeated attempts to clone full-length or partial genome components different from the described above. This was done using the above-mentioned set of restriction enzymes as well as other 6-bp restriction enzymes whose recognition sequences were not present in the cloned genome components (data not shown). Also, *in silico* digestion analysis of cloned begomovirus and deltasatellite (see next section) sequences produced a RCA-RFLP pattern from samples 705 and 706 similar to those experimentally obtained (Figure 4). This analysis supports that no additional begomovirus genome components are present in an amplifiable amount in these samples. Taking together, these results suggested the possibility that CoYVCUV could be a monopartite New World begomovirus.

To assess the putative monopartite nature of CoYVCUV, a dimeric clone obtained for its DNA-A component was agroinoculated alone or in combination with a dimeric clone of DesLDV DNA-B in *Nicotiana benthamiana* and *Corchorus olitorius* plants. In four independent experiments, tissue printing hybridization of apical leaves showed that CoYVCUV was infectious in *N. benthamiana*, all the inoculated plants becoming infected (Table 4 and Supplementary Figure 2A) although they remained asymptomatic (data not shown). This included the two experiments in which CoYVCUV DNA-A was co-inoculated with DesLDV DNA-B, whose replication was not supported based on the tissue printing hybridization analysis (Table 4 and Supplementary Figure 2A). In contrast, *C. olitorius* plants were not infected in any of the four independent experiments (Table 4 and Supplementary Figure 2). Biolistic inoculation of *C. olitorius* plants was also assayed with CoYVCUV DNA-A for which RCA products obtained from circularized monomer genomes were employed. In two independent experiments, *C. olitorius* was not infected as determined by tissue printing hybridization analysis (Table 4 and Supplementary Figure 2B). However, about 50% of the *N. benthamiana* plants inoculated in the same experiments were infected (Table 4 and Supplementary Figure 2B) but remained asymptomatic.

**TABLE 4:** Infectivity of CoYVCUV in several plant species.

Inoculation method	Virus component	Plant	No. infected plants/No. inoculated plants	
			Expt. 1	Expt. 2
Agroinoculation	CoYVCUV	*N. benthamiana*	12/12	12/12
	CoYVCUV + DesLDV DNA-B	*N. benthamiana*	12/12*	12/12*
	CoYVCUV	*C. olitorius*	0/12	0/8
	CoYVCUV + DesLDV DNA-B	*C. olitorius*	0/12	0/8
	CoYVCUV	Zucchini	0/12	0/12
	CoYVCUV	Common bean	5/12	8/12
	CoYVCUV	Pepper	0/12	0/12
	CoYVCUV	Tomato	0/12	0/12
Biolistics	CoYVCUV	*N. benthamiana*	5/12	6/12
	CoYVCUV	*C. olitorius*	0/10	0/10

* Only CoYVCUV was detected in the coinoculated plants.

**FIGURE 5:**
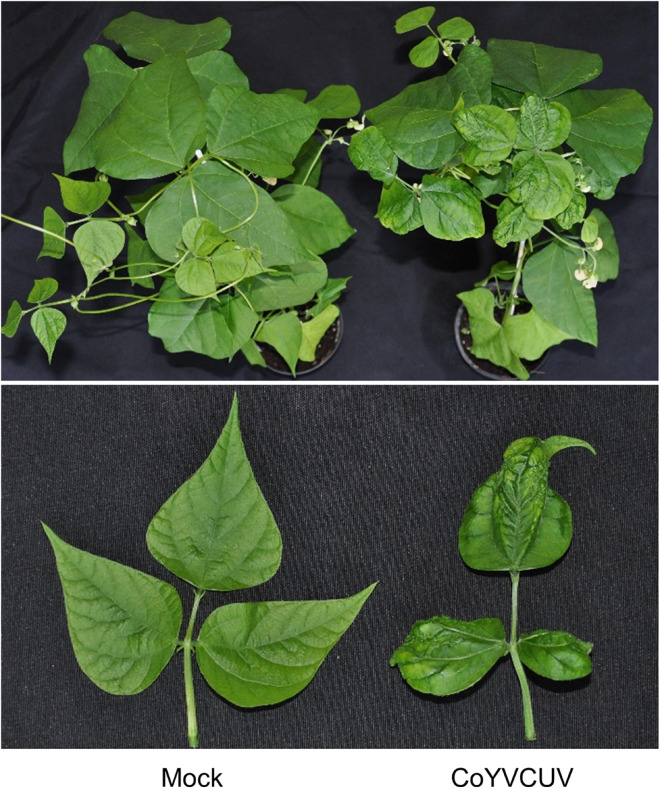
Symptoms of leaf crinkle and mild yellowing developed in common bean plants agroinoculated with Corchorus yellow vein Cuba virus (CoYVCUV) DNA-A. Mock-inoculated controls are shown at the left of each panel.

To further confirm the monopartite nature of CoYVCUV and to get insight on its possible host range, several plant species were inoculated with the CoYVCUV infectious clone. Two independent assays showed that CoYVCUV DNA-A was infectious in common bean, accumulating at detectable levels in apical leaves (Table 4 and Supplementary Figure 2A), but not in tomato, pepper and zucchini. Symptoms developed in common bean plants consisted of leaf crinkle and mild yellowing (Figure 5). These results confirm that CoYVCUV is a monopartite New World begomovirus.

### Two Deltasatellites, One Representing a Novel Species, Are Associated With the Begomoviruses Infecting *Corchorus siliquosus*

Eighteen deltasatellite molecules were cloned and sequenced from three of the five *C. siliquosus* samples showing yellow vein symptoms analyzed in this work (Table 2). Identification of such molecules as deltasatellites was based on their size (665–692 nt) and genome organization, including the features common to all deltasatellites: a small region with some sequence identity to the conserved region present in the betasatellites, an A-rich sequence, a predicted stem-loop structure containing the non-anucleotide TAATATTAC, and a predicted secondary stem-loop. Pairwise comparison between these deltasatellites and all the deltasatellite sequences available in GenBank showed that the deltasatellites from *C. siliquosus* can be readily separated into two groups consisting of those molecules isolated from sample 680 (*n* = 3, 99.7–100% nucleotide identity between them, cloned as *Sac*I fragments, MF773917-MF773919) and samples 704 and 705 (*n* = 15, 96.4–100% nucleotide identity between them, cloned as *Hin*dIII fragments, MF773920-MF773934), respectively. Nucleotide identity between isolates from both groups are only 73.0–74.9%. According to the proposed 91% deltasatellite species demarcation criteria (Briddon et al., 2016), the deltasatellites isolated in this work belong to two different species. The three deltasatellites isolated from sample 680 belong to the species *Tomato yellow leaf distortion deltasatellite 2*, as having the highest nucleotide identity of 97.4% with the only previously known isolate of this species (KU232893) (Fiallo-Olivé et al., 2016). The 15 deltasatellites isolated from samples 704 and 705 showed the highest nucleotide identity (74.4–75.7%) with the five isolates of *Sida golden yellow vein deltasatellite 2* (JN819490-JN819494) (Fiallo-Olivé et al., 2012), thus indicating that they constitute a novel deltasatellite species. Based on the presence of DesLDV in both samples where the novel deltasatellite was found, the name *Desmodium leaf distortion deltasatellite* (DesLDD) is proposed for this species.

**FIGURE 6:**
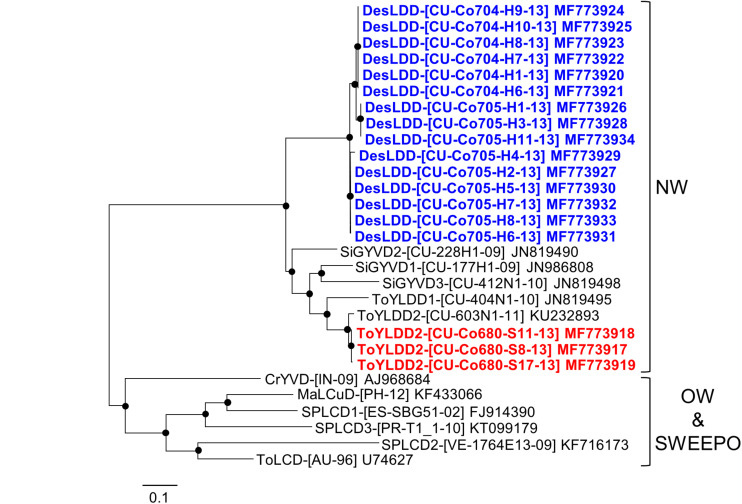
Phylogenetic tree illustrating the relationships of Desmodium leaf distortion deltasatellite (DesLDD) (bold in blue) and tomato yellow leaf distortion deltasatellite 2 (ToYLDD2) (bold in red) isolates obtained in this work with representative isolates of other deltasatellite species. The tree was constructed by the maximum-likelihood method (1000 replicates) with the MEGA 7 program using the best fit model, T92 + G. CrYVD, Croton yellow vein deltasatellite; MaLCuD, Malvastrum leaf curl deltasatellite; SiGYVD1, Sida golden yellow vein deltasatellite 1; SiGYVD2, Sida golden yellow vein deltasatellite 2; SiGYVD3, Sida golden yellow vein deltasatellite 3; SPLCD1, sweet potato leaf curl deltasatellite 1; SPLCD2, sweet potato leaf curl deltasatellite 2; SPLCD3, sweet potato leaf curl deltasatellite 3; ToLCD, tomato leaf curl deltasatellite; ToYLDD1, tomato yellow leaf distortion deltasatellite 1; ToYLDD2, tomato yellow leaf distortion deltasatellite 2. Major deltasatellite clusters associated with bipartite New World begomoviruses (NW) and monopartite Old World begomoviruses and sweepoviruses (OW and SWEEPO) are indicated. The bar below the tree indicates the number of nucleotide substitutions per site. Nodes with bootstrap values higher than 50% are marked with filled circles.

Phylogenetic analysis of all deltasatellites known to date, including those described in this work, showed two main clusters (Figure 6). One of these clusters contains deltasatellites associated with bipartite New World begomoviruses, Sida golden yellow vein deltasatellite 1 (SiGYVD1), SiGYVD2, SiGYVD3, tomato yellow leaf distortion deltasatellite 1 (ToYLDD1), ToYLDD2 and the novel deltasatellite described in this study, DesLDD. All DesLDD isolates clustered together and are separated from the rest of New World deltasatellites, supporting that this deltasatellite constitutes a novel species. No recombination events were detected in the genomes of the deltasatellites described here.

### Desmodium Leaf Distortion Deltasatellite Is Replicated by the Bipartite Desmodium Leaf Distortion Virus but Not by the Monopartite Corchorus Yellow Vein Cuba Virus

Agroinoculation experiments were carried out to asses which begomovirus could act as a helper for DesLDD. For this, a dimeric clone obtained for DesLDD was co-inoculated on *N. benthamiana* and *C. olitorius* plants with dimeric clones of DesLDV DNA-A and DNA-B or of CoYVCUV. As it has been stated above, DesLDV and CoYVCUV did not infect *C. olitorius* in two different experiments. Thus, as expected, DesLDD did not infect this plant. In contrast, both begomoviruses were able to infect *N. benthamiana* (Table 5). In this host, tissue printing hybridization of petiole cross-sections showed that all agroinoculated plants became infected with DesLDV (DNA-A plus DNA-B) or CoYVCUV (Supplementary Figure 3). In two independent experiments, DesLDD replicated and accumulated only in the presence of DesLDV (50% of the plants in both experiments) but not of CoYVCUV (Table 5 and Supplementary Figure 3), thus showing that the former acts as the helper virus.

**TABLE 5:** Infectivity of Desmodium leaf distortion deltasatellite (DesLDD) in the presence of Corchorus yellow vein Cuba virus (CoYVCUV) and Desmodium leaf distortion virus (DesLDV).

Plant species	Helper virus components	Deltasatellite	No. infected plants/No. agroinoculated plants			
			Expt. 1		Expt. 2	
			Virus	Deltasatellite	Virus	Deltasatellite
*N. benthamiana*	CoYVCUV	None	12/12	0/12	12/12	0/12
	CoYVCUV	DesLDD	12/12	0/12	11/12	0/12
	DesLDV A + B	None	12/12	0/12	12/12	0/12
	DesLDV A + B	DesLDD	12/12	6/12	12/12	6/12
	None	None	0/12	0/12	0/12	0/12
*C. olitorius*	CoYVCUV	None	0/12	0/12	0/8	0/8
	CoYVCUV	DesLDD	0/12	0/12	0/8	0/8
	DesLDV A + B	None	0/12	0/12	0/8	0/8
	DesLDV A + B	DesLDD	0/12	0/12	0/8	0/8
	None	None	0/12	0/12	0/8	0/8

**FIGURE 7:**
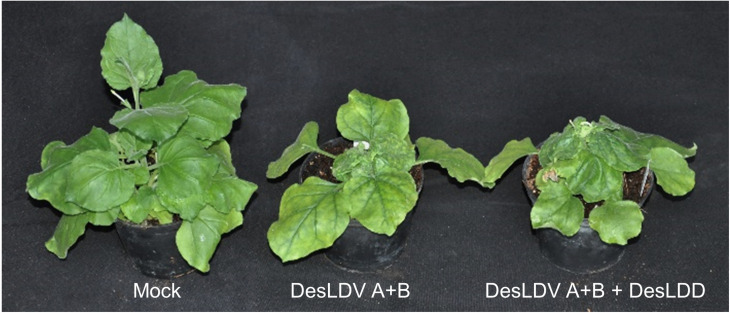
Symptoms of leaf distortion and a significant reduction in plant growth developed in *Nicotiana benthamiana* plants agroinoculated with Desmodium leaf distortion virus (DesLDV) DNA-A and DNA-B, alone or in combination with Desmodium leaf distortion deltasatellite (DesLDD). Mock-inoculated control is shown at the left of the photograph.

*N. benthamiana* plants infected with DesLDV showed symptoms of leaf distortion and a significant reduction in plant growth (Figure 7). The presence of DesLDD did not affect the symptoms caused by DesLDV (Figure 7). The fact that DesLDV is the helper virus of this deltasatellite supports the proposal to name the novel species *Desmodium leaf distortion deltasatellite*.

## Discussion

In this work, a complex of begomoviruses and associated deltasatellites has been characterized from *C. siliquosus* plants showing yellow vein symptoms in Cuba. Isolates of two New World begomovirus species, *Desmodium leaf distortion virus* and *Corchorus yellow vein Cuba virus* have been cloned and sequenced. DesLDV (DNA-A and DNA-B) was found in single infection in several samples, whereas CoYVCUV (DNA-A) was found only in mixed infection with DesLDV. DNA-A from both begomoviruses had the typical genome organization of bipartite New World begomoviruses, but despite repeated attempts, a DNA-B could not be detected for CoYVCUV. Thus, the question remained whether CoYVCUV could be a monopartite begomovirus or it would utilize the DNA-B of DesLDV. Although CoYVCUV DNA-A and DesLDV DNA-B have different iterons, the possibility of forming pseudorecombinants cannot be ruled out. A few cases are known of both New World and Old World begomovirus pairs that form viable pseudorecombinants, at least experimentally, even if their iterons are not identical (Andrade et al., 2006; Chakraborty et al., 2008). Agroinoculation assays carried out in this work showed that (i) CoYVCUV DNA-A alone was infectious in *N. benthamiana* and common bean plants and (ii) DesLDV DNA-B is not replicated in the presence of CoYVCUV DNA-A in *N. benthamiana* plants. Although DNA-As of some bipartite begomoviruses have been shown to be able to infect *N. benthamiana* (Fiallo-Olivé et al., 2016), the fact that CoYVCUV DNA-A was able to infect common bean definitely revealed the monopartite nature of this begomovirus. Although comparison of experimental RCA-RFLP data and *in silico* digestion analysis suggested that no additional begomovirus genome components are present in the studied samples, it does not escape our attention that a deep sequencing approach would have precisely defined if other viruses and/or subviral molecules were present at low concentration.

New World begomoviruses are classically bipartite (Brown et al., 2015; Zerbini et al., 2017). However, in 2011 a monopartite begomovirus native to the Americas was described infecting tomato plants in Peru and Ecuador (Márquez-Martín et al., 2011; Melgarejo et al., 2013; Sánchez-Campos et al., 2013). Later, four additional monopartite begomoviruses have been described from the Americas, all of them infecting tomato: tomato latent virus (TLV) in Cuba (Fuentes et al., 2016), tomato mottle leaf curl virus and tomato leaf curl purple vein virus in Brazil (Vu et al., 2015; Macedo et al., 2018), and tomato twisted leaf virus in Venezuela (Romay et al., 2019). TLV is in fact a recombinant consisting of a moiety from the monopartite Old World tomato yellow leaf curl virus (introduced into the Americas in the early 1990s) and a moiety from an unidentified bipartite New World begomovirus DNA-A (Fuentes et al., 2016). The results obtained in this work expands the number of New World begomoviruses whose monopartite nature has been shown experimentally to six, which is a very low number considering that there are about 170 New World begomovirus species recognized to date. It is generally believed that the first begomoviruses were monopartite (Briddon et al., 2010) and that the bipartite genome likely evolved before continental drift because bipartite begomoviruses occur in the Old and New World (Rojas et al., 2005). It has been suggested that the low number of indigenous monopartite begomoviruses in the New World could be explained by the predominance of bipartite progenitors in the areas of Pangea that gave rise to the Americas or by a later introduction (Melgarejo et al., 2013). The emergence of monopartite begomoviruses infecting tomato in the New World has been hypothesized to represent an evolutionary process which could have occurred due to a combination of factors including: (i) the extensive cultivation of this crop in which monopartite begomoviruses could evolve on their own, (ii) the existence of a local progenitor bipartite begomovirus in wild or cultivated plants, and (iii) the introduction of this local progenitor begomovirus into tomato crops by the invasive and polyphagous Middle East-Asia Minor 1 species (formerly B biotype) of the *Bemisia tabaci* complex (Melgarejo et al., 2013). In contrast to the rest of monopartite New World begomoviruses, CoYVCUV was not found infecting tomato but a wild malvaceous plant and tomato plants could not be experimentally infected by agroinoculation either. Thus, this could represent an additional evolutionary path in generating monopartite New World begomoviruses which could emerge in cultivated plants. Also, the facts that common bean became experimentally infected by CoYVCUV and showed symptoms of leaf crinkle and mild yellowing are relevant since this important crop could be threatened by this begomovirus in Cuba.

Since we were not successful in obtaining *C. siliquosus* seeds, *C. olitorius*, one of the two cultivated species in the genus *Corchorus*, was used in the agroinoculation and biolistics assays. *C. olitorius* was not infected by DesLDV or CoYVCUV, but it should be mentioned that this species is only distantly related to *C. siliquosus* (Benor, 2018).

Other partners in the viral complex found in *C. siliquosus* were subviral molecules, deltasatellites, which are the smallest DNA satellites associated with begomoviruses known to date. Deltasatellites have been previously found associated with two bipartite New World begomoviruses in Cuba, Sida golden yellow vein virus infecting *M. coromandelianum* and tomato yellow leaf distortion virus infecting *S. micranthum* (Fiallo-Olivé et al., 2012, 2016). Here, we have described the presence of two deltasatellites in a third malvaceous plant species, *C. siliquosus*, also from Cuba. One of these deltasatellites, Desmodium leaf distortion deltasatellite (DesLDD), constitutes a novel species, the twelfth of the genus *Deltasatellite*. This finding extends the ranges of natural hosts and helper viruses for these subviral molecules only recently recognized as a novel class of DNA satellites included in the family *Tolecusatellitidae* (Lozano et al., 2016; Adams et al., 2017).

Agroinoculation assays also showed that the novel deltasatellite described here, DesLDD, accumulated in *N. benthamiana* plants in the presence of DesLDV, but not CoYVCUV. Also, the presence of the deltasatellite did not modify the symptoms of leaf distortion and significant reduction in plant growth caused by DesLDV. This result is in agreement with those obtained with the two New World deltasatellites for which infectious clones were available to date, tomato yellow leaf distortion deltasatellite 2 and Sida golden yellow vein deltasatellite 1. Neither of the deltasatellites influenced the symptoms caused by their natural helper begomoviruses in *N. benthamiana* or in their natural hosts (Fiallo-Olivé et al., 2016). In contrast, deltasatellites associated with sweet potato leaf curl virus, a sweepovirus, usually reduced symptom severity caused by its natural helper virus and by tomato leaf curl virus, a monopartite Old World begomovirus (Hassan et al., 2016).

Recombination analysis of representative isolates of the two begomoviruses described in this study showed the recombinant nature of both DNA-As. With the exception of cabbage leaf curl virus (Florida isolate) (Abouzid et al., 1992), all begomoviruses involved in the detected recombination events infect wild plants (*C. siliquosus*, *Desmodium glabrum*, *Jatropha* sp., *Dalechampia* sp., and *Boerhavia diffusa*) in countries from the Caribbean and surrounding areas, from Venezuela to Florida (Hernández-Zepeda et al., 2007, 2009; Fiallo-Olivé et al., 2013; Polston et al., 2014; Melgarejo et al., 2015).

In this work, an additional begomovirus-deltasatellite complex has been characterized in a wild malvaceous plant, with known and novel partners which (i) have been shown to have interacted via recombination with other begomoviruses infecting both cultivated and wild plants and (ii) are able to infect new crops as common bean. In addition, the discovery of a deltasatellite that constitutes a novel species increases the complexity of a recently established, and still poorly understood, class of DNA satellites. This in an example which illustrates the phenomenon of emergence of plant viruses at the interface between natural vegetation and cultivated plants, which can cause outbreaks in nearby crops as well as in new areas driven by whitefly transmission, climate change and global trade.

## Data Availability Statement

The datasets generated for this study can be found in the online repositories. The names of the repository/repositories and accession number(s) can be found here: https://www.ncbi.nlm.nih.gov/genbank/, MF773881–MF773934.

## Author Contributions

EF-O and JN-C collected the *Corchorus siliquosus* leaf samples in the frame of the Ibero-American Network on Integrated Management of Vegetable Virus Diseases (CYTED 111RT0433), analyzed the data, and wrote the manuscript. EF-O and JN-C conceived the experiments that were performed by EF-O. Both authors contributed to the article and approved the submitted version.

## Conflict of Interest

The authors declare that the research was conducted in the absence of any commercial or financial relationships that could be construed as a potential conflict of interest.

## Footnotes

1 https://blast.ncbi.nlm.nih.gov/Blast.cgi
