# Supplementary material for: Molecular and Biological Characterization of a New World Mono-/Bipartite Begomovirus/Deltasatellite Complex Infecting Corchorus siliquosus
Source: Front Microbiol. 2020 Jul 23;11:1755. doi: 10.3389/fmicb.2020.01755 (PMC7390960; doi:10.3389/fmicb.2020.01755)
Supplement: Supplementary file 1 [file Data_Sheet_1.PDF]

SUPPLEMENTARY TABLE 1 | Cloning strategy to obtain the infectious dimer plasmids of CoYVCUV, DesLDV (DNA-A and DNA-B) and DeLDD.

**DNA component**	**Monomer enzyme[1]**	**Dimer enzyme[2]**	**Binary vector enzymes[3]**	**Infectious dimer plasmid**
CoYVCUV	*Sac*I	*Eco*RI	*Spe*/*Hind*III	pC0380dimCoYVCUV
DesLDV DNA-A	*Sac*I	*Pst*I	*Spe*/*Hind*III	pC0380dimDesLDV-A
DesLDV DNA-B	*Sac*I	*Eco*RI	*Spe*/*Hind*III	pC0380dimDesLDV-B
DesLDD	*Hind*III	*Hind*III	*Eco*RI/*Sal*I	pC0380dimDesLDD

[1]*Enzyme used to clone the virus or satellite monomers in the vectors pGEM-T Easy or pBluescriptII SK(+).*
[2]*Enzyme used to clone the virus or satellite dimers in the vector pBluescriptII SK(+).*
[3]*Enzymes used to clone the virus or satellite dimers in the binary vector pCAMBIA0380.*

SUPPLEMENTARY TABLE 2 | List of primers designed to amplify by PCR the DNA fragments of each begomovirus and deltasatellite genomic component to prepare the probes used in this study.

**Probe**	**Primer name**	**Primer sequence (5´- 3´)**
CoYVCUV	MA2807 (FW)	CTCTTGCATTTCTTCCGGGACG
	MA2808 (RV)	GGAAAGTTCAAATGCCAGAATAAC
DesLDV DNA-A	MA2805 (FW)	GTCCGGCACGTTAGTGAAAGAG
	MA2806 (RV)	GAAGCTCTTCAGATGCTTCAAAAC
DesLDV DNA-B	MA2550 (FW)	CGAGCCCAAGTTGACAAGCCAAACC
	MA2551 (RV)	GGATGCTTTAGACATGGTATCGGAC
DesLDD	MA2554 (FW)	GTCCGTGTCACTTCTTTATCTCCGC
	MA2555 (RV)	CTTAGCTACGGCGGAGCTAAGGCTG

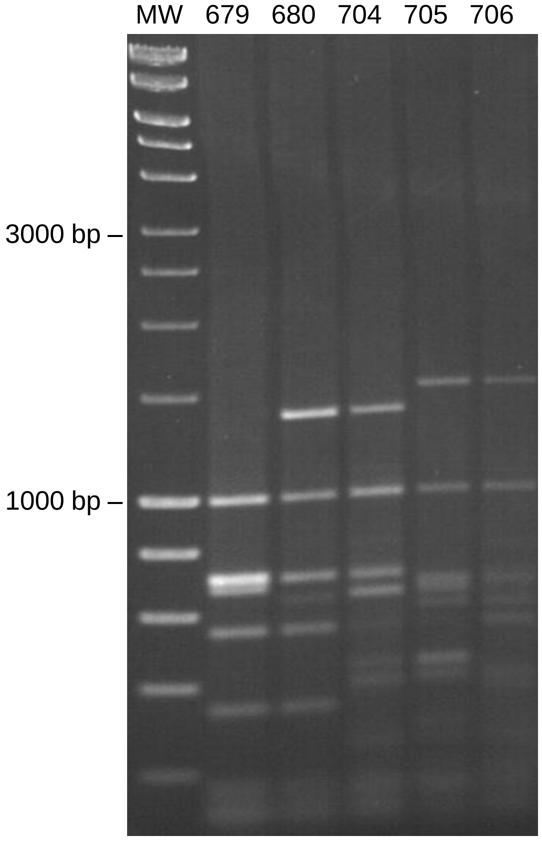

SUPPLEMENTARY FIGURE 1. Restriction fragment length polymorphism (RFLP) analysis performed by digestion of rolling circle amplification (RCA) products obtained from *Corchorus siliquosus* plant DNA extracts (samples 679, 680, 704, 705 and 706) with the restriction enzyme *Hpa*II revealed on a 1.5% agarose gel. MW: molecular weight marker (HyperLadder 1kb, Bioline).

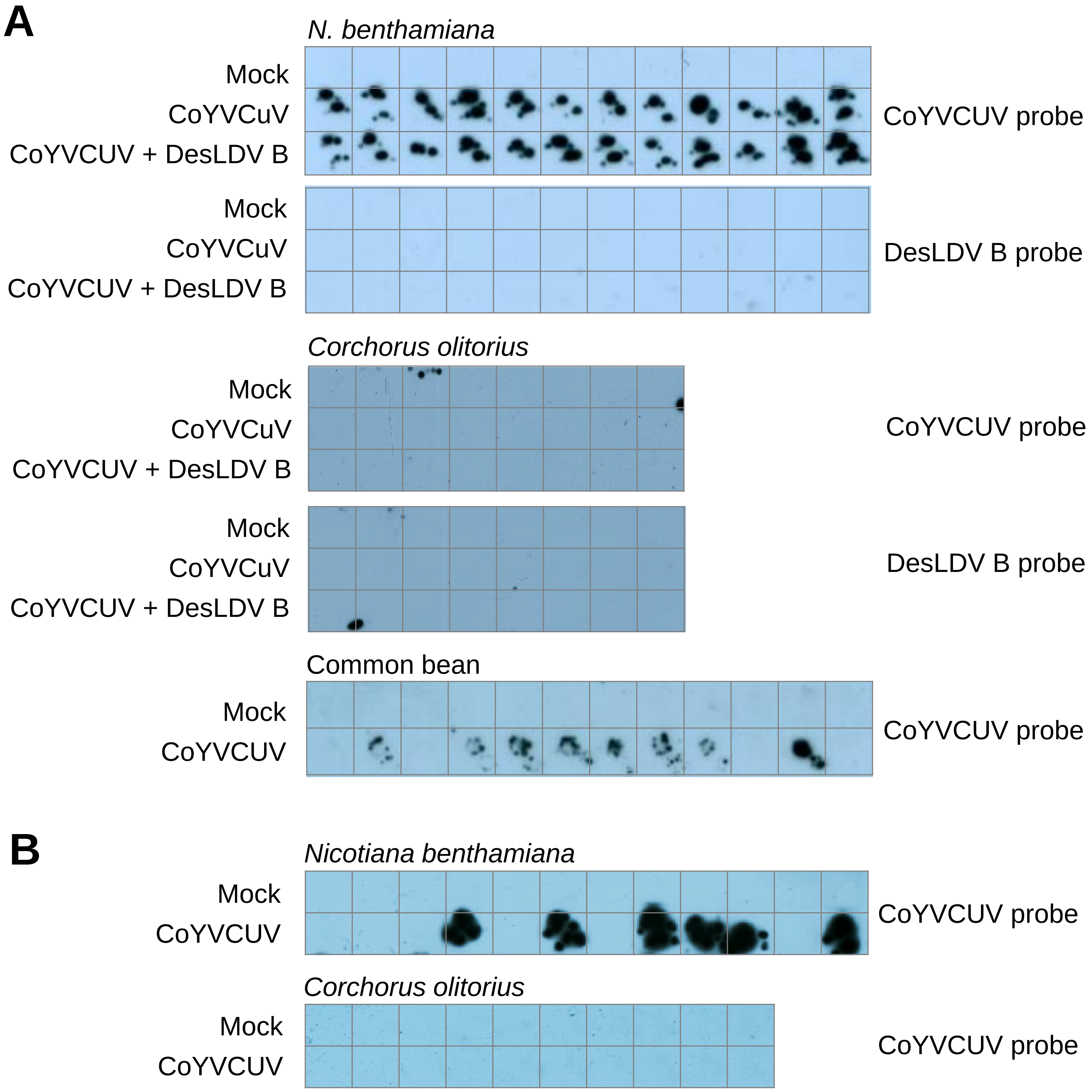

SUPPLEMENTARY FIGURE 2. Inoculation experiments with Corchorus yellow vein Cuba virus (CoYVCUV) DNA-A (alone or in combination with DesLDV DNA-B). CoYVCUV DNA-A and DesLDV DNA-B specific digoxigenin-labelled DNA probes were used to analyze agroinoculated *Nicotiana benthamiana, Corchorus olitorius* and common bean plants **(A)** or biolistically inoculated *N. benthamiana* and *C. olitorius* plants **(B)** after tissue printing on nylon membranes. Tissue prints of mock-inoculated plants were included as negative controls. Results correspond to Expt. 2 in Table 4.

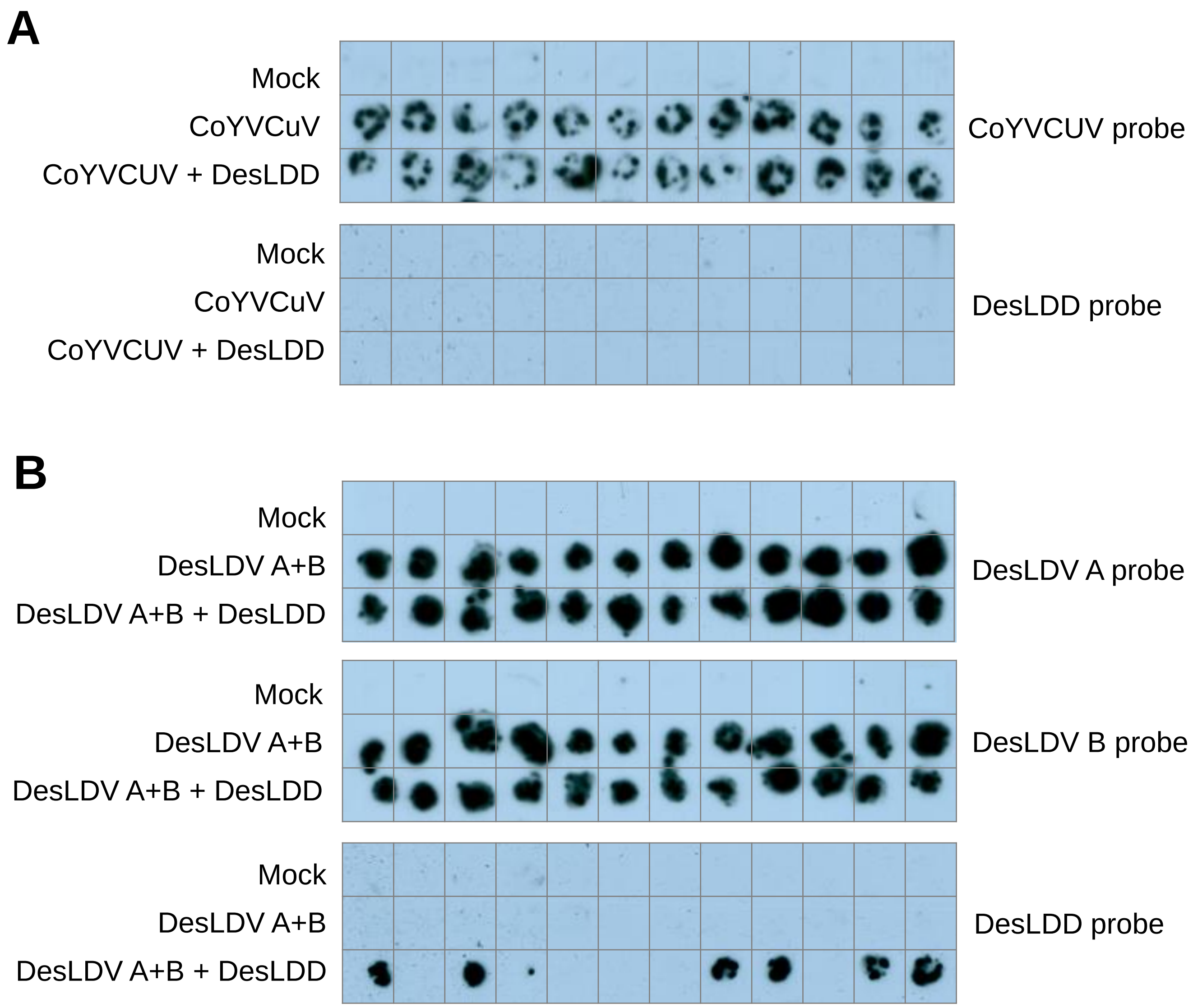

SUPPLEMENTARY FIGURE 3. Agroinoculation experiments with Desmodium leaf distortion deltasatellite (DesLDD) in the presence of Corchorus yellow vein Cuba virus (CoYVCUV) DNA-A **(A)** or Desmodium leaf distortion virus (DesLDV) DNA-A and DNA-B **(B)**. CoYVCUV DNA-A, DesLDV DNA-A, DesLDV DNA-B and DesLDD specific digoxigenin-labelled DNA probes were used to analyze agroinoculated *Nicotiana benthamiana* plants after tissue printing on nylon membranes. Tissue prints of mock-inoculated plants were included as negative controls. Results correspond to Expt. 2 in Table 5.
